# Reply to Loh: Evaluating anger as a mediator of criticism’s effect on social media engagement using the interventional effects approach

**DOI:** 10.1073/pnas.2529782122

**Published:** 2025-12-15

**Authors:** Jonathan Y. Lee

**Affiliations:** ^a^Department of Psychiatry and Behavioral Science, Stanford University School of Medicine, Stanford, CA 94305

In two online survey experiments, I found evidence suggesting that institutional criticisms featuring integrity-based narratives (as opposed to competence-based ones) generated dramatically more anger and social media engagement preferences likely to promote viral spread and exacerbate preexisting institutional politicization and issue polarization (1). In mediation analyses preregistered as exploratory, I used single-mediator models which found evidence suggesting that anger fully mediated certain highlighted effects of exposure to integrity-based criticism on the strength of social media engagement preferences. In a commentary, Loh (2) raises valid concerns about the use of single-mediator models when evaluating multiple mediators and recommends the use of an interventional effects approach to multiple mediation analysis to account for the potential for multiple mediators to have confounding effects on each other (3, 4). Using this approach, he reanalyzed my data and found that “[…] none of the putative mediators (four emotional responses) fully mediated any effect. All the mediated effect estimates were closer to zero, or even in the opposite direction, than originally suggested” (2).

I examined Loh’s (2) results and noticed that in addition to his indirect effect estimates differing from those of my original analysis, so did his total mediation effect estimates, which would not be expected from the sole shift from single-mediator to multiple-mediator models. Explaining this discrepancy, his reanalysis code revealed an important difference in how we constructed the “Absolute Value of Social Media Engagement Preferences Indices,” the measure of respondents’ strength of social media engagement preferences. While I used

$$\begin{array}{c} Abs\left(Downvote\;vs.Upvote\;measure\right)\\ +Abs\left(Flag\;vs.Share\;measure\right)\\ +Abs\left(Block\;vs.Follow\;measure\right). \end{array}$$

He used:

$$\begin{array}{c} Abs(\left(Downvote\;vs.Upvote\;measure\right)\\ +\left(Flag\;vs.Share\;measure\right)\\ +\left(Block\;vs.Follow\;measure\right)). \end{array}$$

This misunderstanding is a result of my failure to clearly describe the construction of these indices in *SI Appendix* (1) (a change to the *SI Appendix* is forthcoming).

**Fig. 1.**
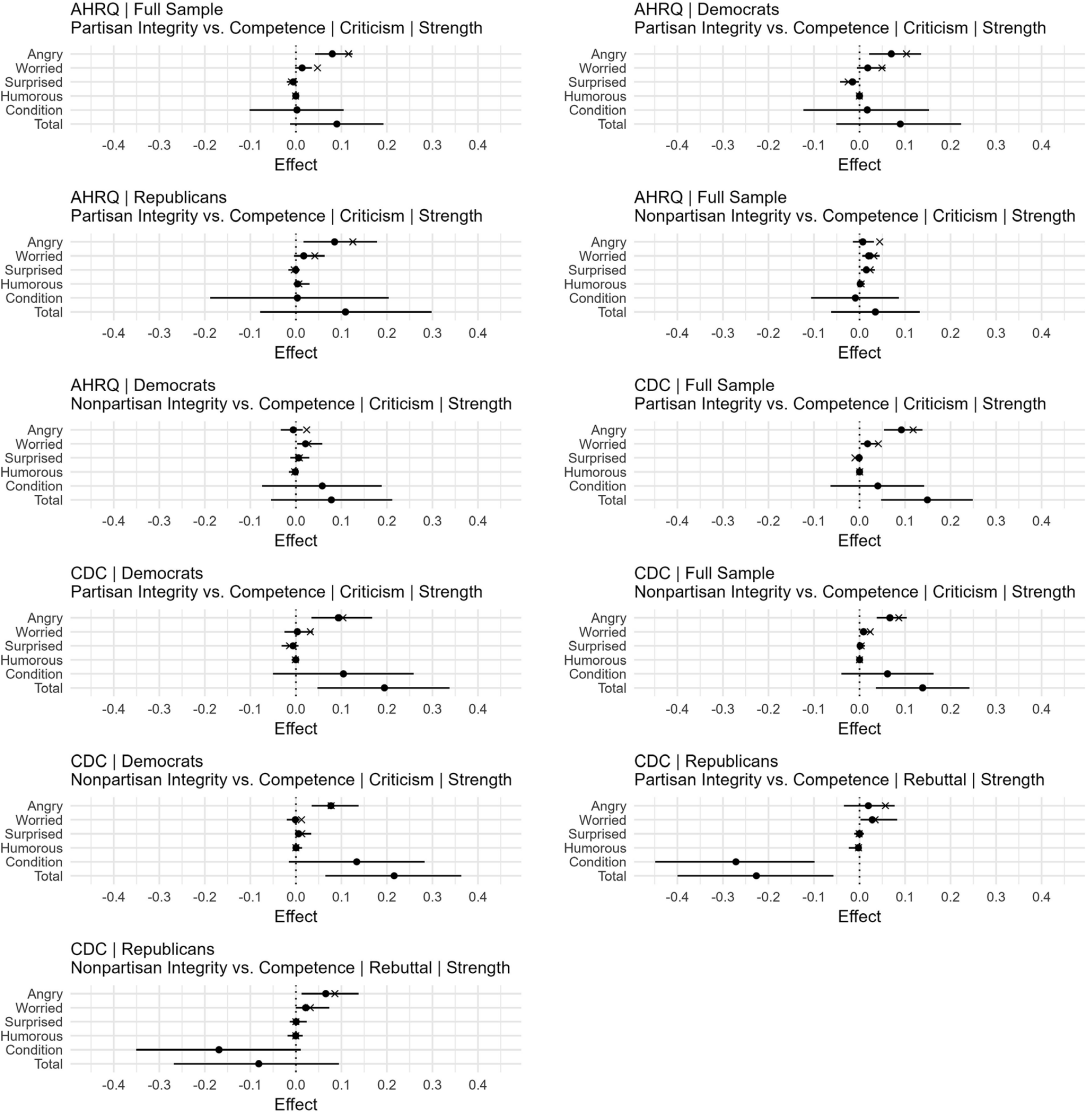
Multiple mediation analyses results: emotions and strength of social media engagement preferences by experiment (AHRQ vs. CDC), sample (All, Republican subgroup, Democrat subgroup), experimental arm comparison (Partisan Integrity vs. Competence or Nonpartisan Integrity vs. Competence), measure (Strength of social media engagement preferences), and social media post (Criticism social media post vs. Rebuttal social media post). The interventional indirect effect point estimates for “Angry,” “Worried,” “Surprised,” and “Humorous” are indicated by filled circles. Horizontal lines represent their 95% Bias-Corrected and Accelerated (BCa) bootstrap CI using 10,000 bootstrap iterations. For comparison, the indirect effect estimates for each emotion using separate single mediation analyses are indicated by x’s. “Condition” refers to the interventional direct effect estimates, while “Total” refers to the interventional total effect estimates.

With the index construction corrected, I replicated Loh’s (2) analysis. As explicitly stated in the original paper (1), I specifically focused on mediation of the strength of social media engagement preferences effects that were considered more statistically meaningful based on their individual CIs and trends replicating across both independent experiments. These results can be seen in Fig. 1.

Having adjusted for multiple mediators, all indirect effect estimates experienced some attenuation after adjusting for the other mediators. Nevertheless, there remained compelling evidence suggestive of anger fully mediating the potential effects of exposure to integrity-based criticisms on the strength of social media engagement preferences. In eight of the eleven plots, the indirect effect estimates of anger remained larger than those of the other emotions, with *P*_unadjusted_-values remaining below 0.05. The *P*_unadjusted_-values of the corresponding direct effect estimates also remained above 0.05. I repeat the same caveat from my original paper: These are exploratory findings that will benefit from replication with larger sample sizes.

I appreciate Loh (2) for raising awareness to a more optimal approach to multiple mediation analysis. I do not take for granted the public service that he and others provide by constructively scrutinizing the statistical methods used in published results.
